# Exploring the spatial patterns of soil salinity and organic carbon in agricultural areas of Lesvos Island, Greece, using geoinformation technologies

**DOI:** 10.1007/s10661-023-10923-5

**Published:** 2023-02-13

**Authors:** Christina Lekka, George P. Petropoulos, Dimitris Triantakonstantis, Spyridon E. Detsikas, Christos Chalkias

**Affiliations:** 1 Department of Geography, Harokopio University of Athens, El. Venizelou 70, Kallithea, 17671 Athens, Greece; 2 Department of Soil Science of Athens, Institute of Soil and Water Resources, Hellenic Agricultural Organization–DIMITRA, 1 Sofokli Venizelou, 14123 Lycovrisi, Attiki Greece

**Keywords:** Soil salinity, Soil organic carbon, Logistic regression, Remote sensing, GIS

## Abstract

The salt-affected soils national map of Greece was recently made available within the initiative of the Global Soil Partnership (GSP) of Food and Agriculture Organization of the United Nations FAO. The present study explores the development of higher resolution soil property maps included in this national scale product adopting a modified version of the FAO methodology and a logistic regression (LR) method based on ground and satellite data. Furthermore, it also investigates the correlation between saline soils and soil organic carbon (SOC) using geospatial analysis methods. The island of Lesvos in Greece has been selected as a case study. A probabilistic model for saline soils in the agricultural land of Lesvos is produced by exploiting geoinformation technologies. As a result, the spatial distribution of saline soils in the croplands of Lesvos was obtained. Indicatively, areas with *p* > 0.80 for the occurrence of saline soils accounting for ∼20% of a total area of 169.51 km^2^ of the croplands in Lesvos. The Nagelkerke *R*^2^ coefficient showed that the probabilistic model interprets 11.3% of the variance of the dependent variable from the independent factors. The model accuracy was assessed adopting the receiver operating characteristic (ROC) curve, which showed a reasonable adaptability with area under curve (equal to 0.73). The methodological approach proposed herein can support decision-making on agricultural land protection and planning activities which are key priority today due to environmental instability, food security, and climate change.

## Introduction

Soil salinity is considered worldwide as one of the most important soil health threats contributing to soil degradation and halting agricultural productivity (Rengasamy, 2016). Soil salinization is a restricting factor of agricultural production, especially in arid and semi-arid areas, as the high concentration of salts affect soils’ quality, vegetation growth, crop diversity, and food production (Abdennour et al., 2020). The development of soil salinity in an area is closely linked to its topographic and climatic characteristics, unsustainable agricultural management practices, and insufficient drainage (Dagar et al., 2019). Moreover, factors such as high temperature and low seasonal precipitation, humidity, and topographical features affect salinization in soil (Tomaz et al., 2020; Hopmans et al., 2021).

Previous research in salt-affected soils has revealed that salinization processes negatively affect a number of key soil properties such as soil structure and quality, microbial biomass, and activity as well as soil organic carbon (SOC) content and its decomposition rates (Wong et al., 2010). The relationship between SOC and soil salinity has been investigated by numerous studies. In salt-affected soils, SOC dynamics are contradictious, as on one hand, salinity hinders plant growth reducing above ground biomass and consequently reducing soil organic matter content (Wong et al., 2006). On the other hand, increased salinity levels hinder soil microbial activity reducing decomposition rates and thus resulting to an increase in SOC content (Setia et al., 2013). In a recent study, Enya et al. (2020) examined the effect of heavy metals on organic matter decomposition in the Upper Mersey estuarine floodplain, in Northwest England and reported significant relationships between soil organic carbon and salinity. The authors also highlighted the impact of electrical conductivity (EC) in soil carbon dynamics and microbial activity in organic matter decomposition. The effects of soil salinity in SOC are exacerbating, especially when salinity is developed in parallel with the adoption of unsustainable management practices such as extensive tillage, use of pesticide and fertilizers, and removal of crop residues. Those are common in Europe’s agricultural land, as increase the mineralization of soil organic matter (SOM) which results to SOC reduction (Lal, 2004)*.*

In the purview of the above, but also due to the urgent need to develop strategies that will help mitigating climate change impacts, it is necessary to develop methods and approaches to detect, monitor, and evaluate the extent of salt-affected soils. This is particularly urgent in rural areas where inappropriate irrigation along with poor drainage and high evapotranspiration rates increases salinization risk. To monitor physical phenomena, such soil salinity, geoinformation technologies, and in particular remote sensing (RS) and geographic information systems (GIS) provide a viable solution due to their advantages over conventional approaches (Srivastava et al., 2019). RS is a non-distractive approach that allows monitoring salt-affected soils at a variety of spatial and temporal extents cost and time effective manner (Abbas et al., 2013; Tsatsaris et al., 2021). The use of RS in mapping and monitoring salt-affected soils has been demonstrated in an array of studies (e.g., Delavar et al., 2020; Suleymanov et al., 2021; Wang et al., 2019). These advantages constitute RS and GIS reliable tools to detect and monitor soil salinity (Aksoy et al., 2022; Gorji et al., 2017a, b). Different combinations of spectral bands and their mathematical transformations, such as principal component analysis (PCA) and satellite radiometric indices, have been used successfully to determine salt-affected soils (e.g., Kumar et al., 2019). Satellite data from multispectral optical sensors such as Landsat TM have a high potential for monitoring the spatiotemporal extent of salts’ accumulation in the top soil (Nguyen et al., 2020; Wang et al., 2019). The combined use of satellite and ground truth data coupled with GIS techniques is the most common approach for mapping the extent of salt-affected soils due to the advantages offered by this synergistic use (Gorji et al., 2019; Taghadosi et al., 2019). A significant milestone in the use of RS, GIS, and modeling approaches for mapping salt-affected soils is the recently launched Global Map of Salt Affected soils (GSAS) by FAO’s working group focusing on soil, Global Soil Partnership (GSP). GSAS modeling approach includes the combination of satellite, climate, geomorphological, geological, and soil legacy data along with the expertise of scientists to map the global extent of salt-affected soils (FAO, 2020).

FAO’s GSAS map indicates that the global extent of salt-affected soils is more than 424 million ha of topsoil (0–30 cm) with ~ 85% of salt-affected topsoil’s are saline, 10% are sodic, and 5% are saline-sodic. More than two-thirds of the global salt affected soils can be found under arid and semi-arid climatic zones (FAO, 2021), such as those of the Mediterranean region which is a global hotspot of salt-affected soils. According to FAO, ~ 25% of irrigated agricultural land is affected by soil salinization (FAO, 2010). In Greece, salt-affected soils are mainly formed in arid or semi-arid irrigated lands and croplands. Lesvos island is one area in Greece where salinization has been studied extensively over the recent years, induced not only due to climate conditions but also due to inappropriate fertilization and irrigation systems in agricultural areas of the island (e.g., Yassoglou & Kosmas, 2002).

In purview of the above, the present study proposes a modified version of the FAO’s model for mapping saline soils that is based on combining field measurements and satellite data, which is evaluated in the crop areas of Lesvos Island in Greece. In particular, the study objectives are (i) to develop a probability model for the spatial distribution of saline soils in cropland areas of Lesvos and (ii) to perform an exploratory analysis investigating the spatial correlation between the distribution of saline soils and the SOC stock contained in those soils. The methodology adopted to satisfy the objectives is developed in a GIS environment and is a modified version of FAO’s recently proposed approach for mapping salt-affected soils.

## Study area

**Fig. 1:**
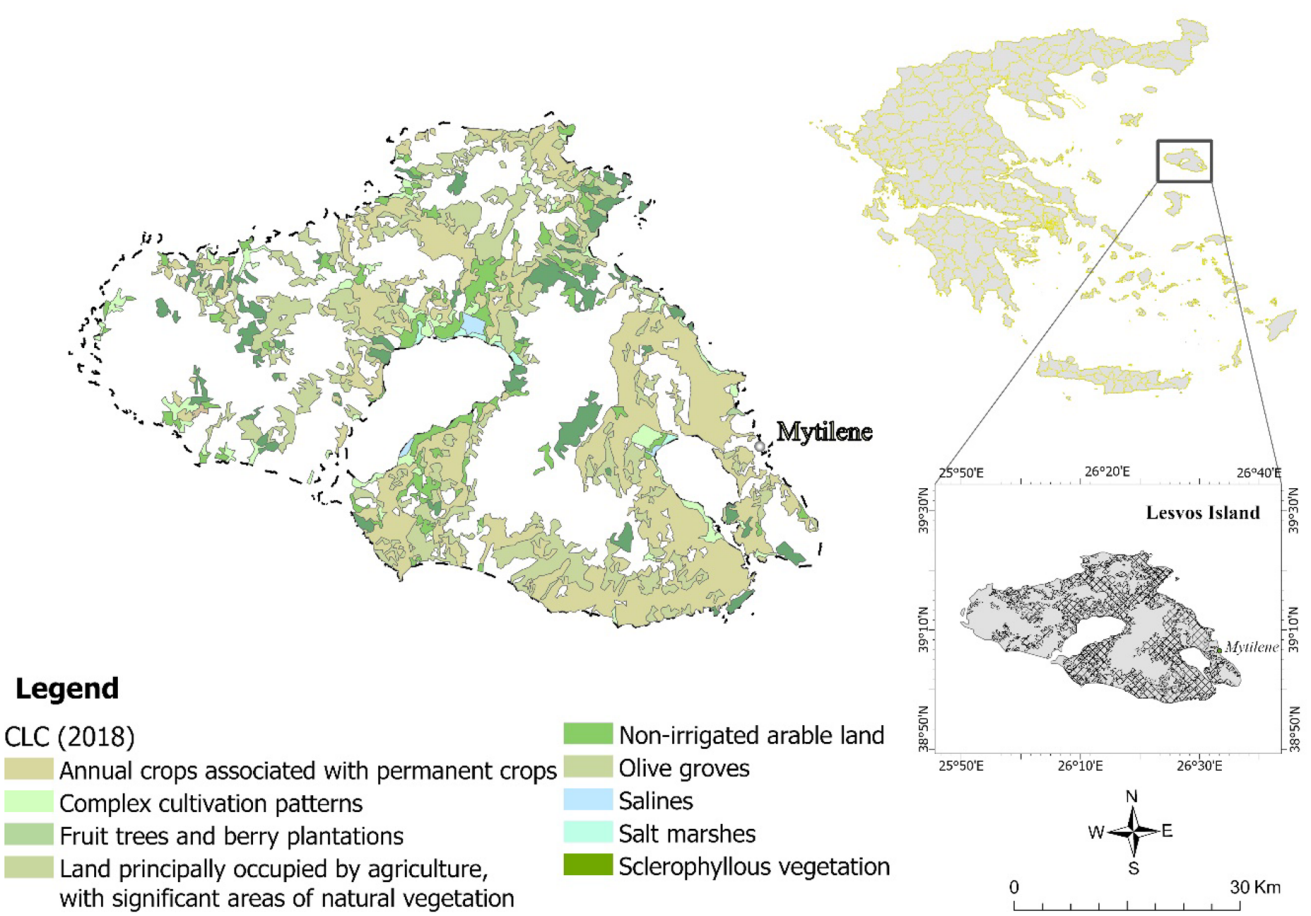
The selected study site location in Greece and the land cover map of Lesvos for croplands according to the Corine Land Cover (CLC) 2018 program

Lesvos island is the third largest island of Greece located in the northeastern part of the Aegean, extending from latitude 39° N to longitude 26° W (Fig. 1). Lesvos flora is particularly diverse ranging from natural grassland, shrublands, and forests to perennial orchards and annual irrigated crops. The island’s climate is characterized by seasonal patterns in rainfall and temperature with dry-thermal summers and humid winters. The average temperature is ~ 17 °C with seasonal variations ranging up to ~ 15 °C. There is a strong spatial pattern with rainfall ranging from 725 mm to the eastern (wetland) to 415 mm to the western (semi-arid) island (Bakker et al., 2005). The xerothermic conditions characterizing the island, combined with the adoption of unsustainable agricultural practices followed by local farmers, create ideal conditions for the development of salt-affected soils. In western Lesvos, where the climatic conditions are more arid, saline soils have been developed leading to the degradation and desertification of the area (Kosmas et al., 1999, 2002).

## Datasets

For this study, a number of geomorphological, climatic, and satellite data were collected and processed, summarized in Table 1, and are briefly described below.

**Table 1:** Datasets used in this study

**Data layers**	**Data type** **(cell size/scale)**	**Data source**
Mean multispectral imagery data and salinity and vegetation indices	Raster grid 30 × 30 m	USGS Landsat 8 Level 2, Collection 2, Tier 1 https://earthexplorer.usgs.gov/
DEM	Raster grid 30 × 30 m	ASTER Earth Explorer–USGS https://earthexplorer.usgs.gov/
Climate parameters mean annual	Raster grid 4 × 4 km	TerraClimate https://www.climatologylab.org/terraclimate.html
Soil dataset with EC_e_ measurements	Polygon and point	Soil data (Mispopolinos, 2015 vía ELGO–DIMITRA)
Corine Land Cover (CLC)	Polygon (scale: 1:100.000)	Corine Land Cover (CLC) 2018 https://land.copernicus.eu/pan-european/corine-land-cover/clc2018
Topsoil Soil Organic Carbon (LUCAS)	Raster grid 500 × 500 m	de Brogniez et al. (2015). European Soil Data Centre (ESDAC) https://esdac.jrc.ec.europa.eu/content/topsoil-soil-organic-carbon-lucas-eu25

### Field measurements

**Fig. 2:**
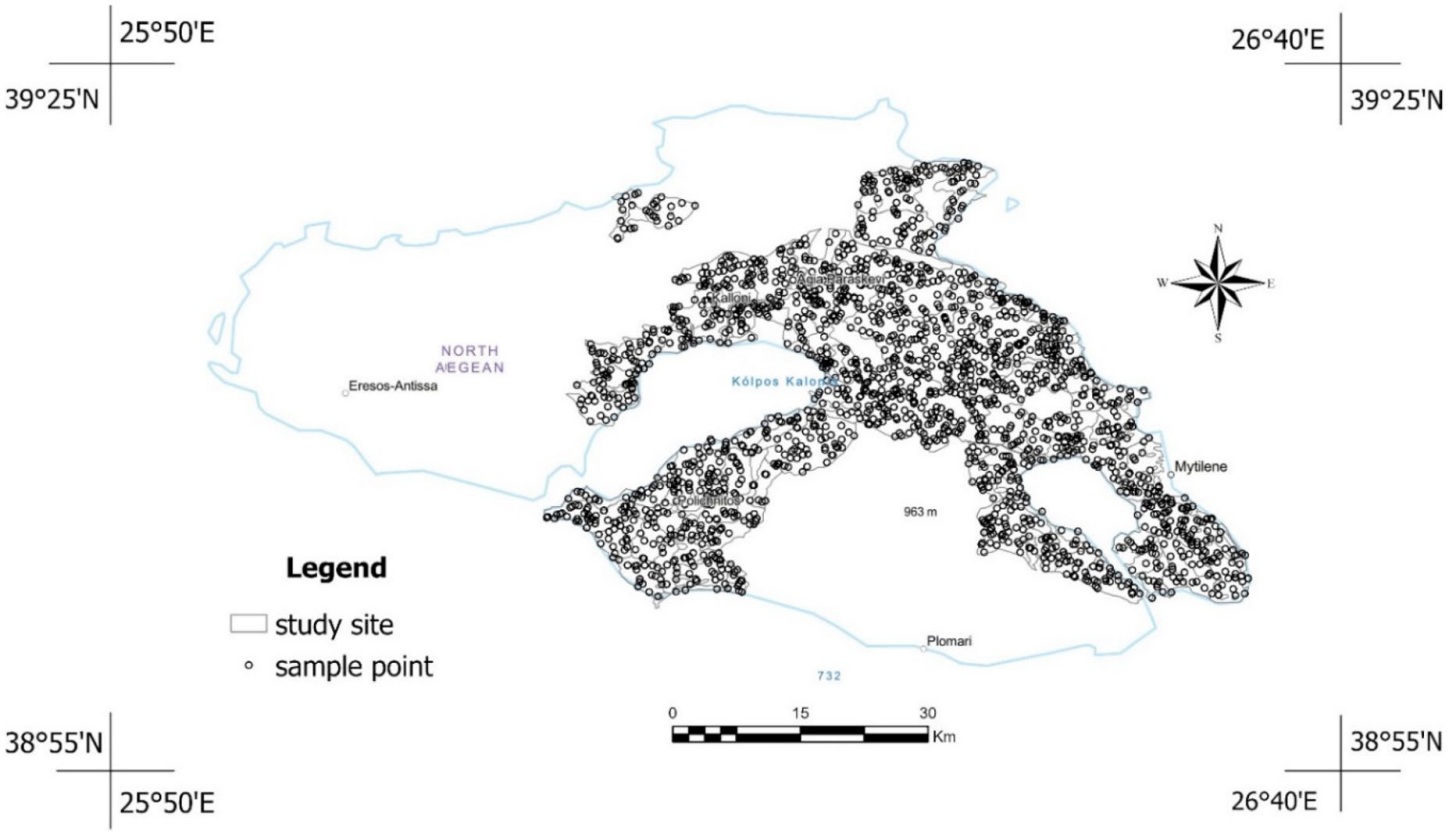
Spatial distribution of available field data within the extent of the study area

The field data used in this study was collected in 2015 in scattered locations of eastern and central Lesvos from topsoil. After their collection, the physical and chemical properties of the soil samples were analyzed using wet-chemistry procedures in a soil laboratory. The electrical conductivity of saturated paste (EC_e)_ contained in the derived dataset was used. The majority of the ECe measurements, with a percentage of 89.7%, in the used soil dataset, were characterized with values higher than 4 dS m^−1^, whereas 7.8% of soils had EC_e_ values from 4 to 8 dS m^−1^. Finally, EC_e_ values ranging from 8 to 15 or higher than 15 dS m^−1^ appear in soils with a percentage of 1.7% and 0.9%, respectively. The soil data used herein was provided by the archive of the Soil Department of Athens–Institute of Soil and Water Resources, ELGO–DIMITRA (Misopolinos, 2015). In Fig. 2 is depicted the location of the used soil samples scattered in areas of eastern and central Lesvos.

### Satellite data

As inputs to the model developed herein, vegetation and salinity indices were estimated using Landsat 8 OLI USGS Level 2 Collection 2 Surface Reflectance (SR), Tier 1 multispectral imagery mean values for 2015 for the island of Lesvos at 30-m spatial resolution. Landsat 8 SR imagery was acquired using Google Earth Engine (GEE), a cloud-based platform that enables cost and time-effective accessing and processing of RS data. Furthermore, the mean, maximum, and minimum temperature (°C) as well as the precipitation values (mm) for 2015, were acquired from Terra Climate at 4-km spatial resolution (Abatzoglou et al., 2018) using GEE. Furthermore, ASTER’s Digital Elevation Model (DEM) of Lesvos was acquired at a spatial resolution of 30 m from the USGS Earth Explorer platform. All datasets were acquired at no cost.

### Soil organic carbon map

Soil organic carbon stock data were obtained at no cost from the European Soil Data Centre (ESDAC) (Panagos et al., 2012). The Topsoil Organic Carbon (LUCAS) map was developed in 2014 with a spatial coverage of 25 member states of the European Union and a spatial resolution of 500 m. These data refer to the estimated reserves of soil organic carbon (SOC) of 0–20-cm soils’ depth, in g C kg^−1^ measurements. A generalized additive model (GAM) was fitted on 85% of the dataset (*R*^2^ = 0.29), using OC content as the dependent variable, while the model accuracy gave an overall *R*^2^ of 0.27 (de Brogniez et al., 2015).

## Methodology

**Fig. 3:**
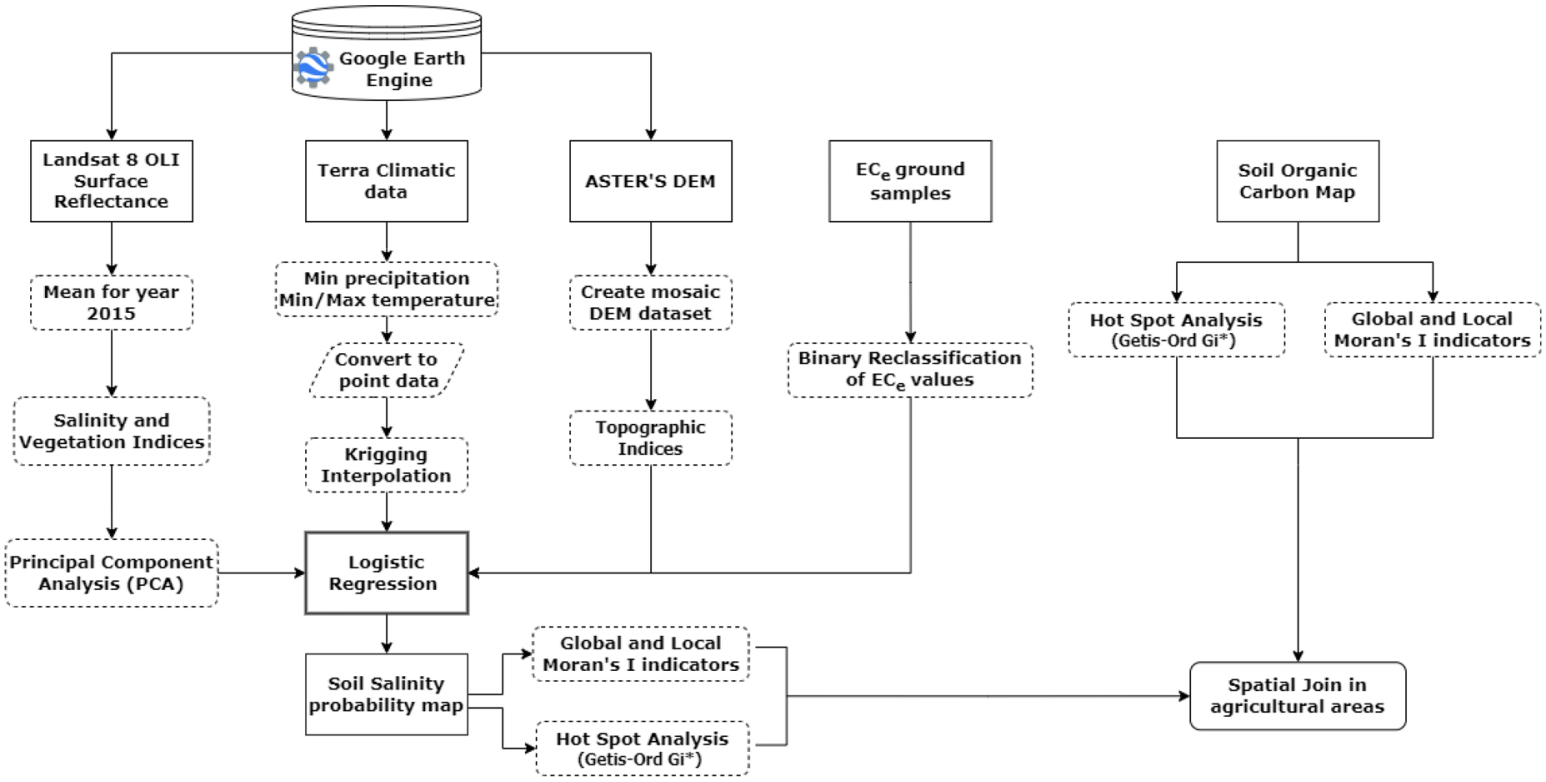
Overview of the methodology adopted herein for estimating the salt-affected soils map and in exploring their relationship with soil organic carbon stock

An overview of the key steps included in the methodology developed to satisfy the study objectives is presented below (Fig. 3). The spatial distribution of salt-affected soils came through the development of the probability model. Furthermore, the relation between salt-affected soils and SOC is examined using indicators of spatial autocorrelation.

### Pre-processing

All the drivers needed for the development of the probability model of salt-affected soils were stored in the spatial database with a defined spatial resolution of 30 × 30 m using the ArcGIS software. The acquired geospatial data was first georectified to a Universal Transverse Mercator (UTM) coordinate system, using the World Geodetic System (WGS) 1984 datum and UTM zone 34.

#### Topographic indices

The DEM of the study area was used to derive a series of topographical indicators, related to the processes of soil salinization. Those included the slope, plan curvature, channel network base level, and topographic wetness index represent terrain attributes that have been previously used for modeling soil salinity (Omuto et al., 2020; Shahrayini & Noroozi, 2022; Salcedo et al., 2022). Those datasets were produced using the basic terrain analysis tool available in QGIS open source software. The produced factors were used as the independent variables in the development of the probability model of salt-affected soils.

#### Climatic parameters

The climatic parameters used in this study refer to the average value of rainfall and maximum/minimum temperature for the year 2015. Due to the lower spatial resolution of the TerraClimate climatic parameters, the ordinary kriging geostatistical interpolation technique was used to downscale the needed climatic data (Davy & Kusch, 2021). Ordinary kriging estimates the value in a specific point based on a linear combination of the measured values surrounding in this location (Srivastava et al., 2019). The variogram determines the weights and provides information about the spatial correlation of the data (Sluiter, 2009). The ordinary kriging method assumes intrinsic stationarity in the distribution of parameters due to an unknown mean. Layers created by centroid values of each pixel in climatic products were obtained so as for applying the interpolation method. Interpolated surface models were created herein for each parameter of min/max temperature and precipitation using the ordinary kriging, where in both cases the spherical variogram model with sector number 4 with 45° offset was used. Statistics calculated using the cross-validation method indicated a root mean square standardized (RMSS) error of 0.93 for annual rainfall, 0.89 and 0.86 for minimum and maximum temperature, respectively. The spatial resolution of interpolated climatic parameters was defined as 30 × 30 m and adjusted to the model rather than the low resolution (4 km) prior to interpolation processes.

#### Vegetation and salinity indices

The satellite vegetation and salinity indices were developed from the Landsat 8 OLI SR satellite imagery. The following satellite indicators developed are SI (1), SI (2), SI (3), SI (4), SI (5), SI (6), NDVI, NDSI, SAVI, SR, CRSI, BI, and VSSI (see Table 2). These indicators are effective and have been used in various studies to monitor and detect salt-affected soils (Abuelgasim & Ammad, 2019; Allbed et al., 2014; Gorji et al., 2019; Nguyen et al., 2020; Scudiero et al., 2017).

**Table 2:** Indices for soil salinity assessment according to the technical manual of SAS mapping in the methodology adopted by FAO (2020)

Indices	Formula	Source
Salinity Index 1	$S\mathrm{I}=\sqrt{\mathrm{Green}\times \mathrm{Red}}$	(Abbas & Khan, 2007)
Salinity Index 2	$S2=\sqrt{\mathrm{Blue}\times \mathrm{Red}}$	(Abbas & Khan, 2007)
Salinity Index 3	$S3=\sqrt{{\mathrm{Green}}^{2}\times {\mathrm{Red}}^{2}}$	(Douaoui et al., 2006)
Salinity Index 4	$S4= \frac{\mathrm{NIR}\;\times\;\mathrm{SWIR}\;-\;{\mathrm{SWIR}}^{2}}{\mathrm{NIR}}$	(Douaoui et al., 2006)
Salinity Index 5	$S5= ^\mathrm{Blue}/_\mathrm{Red}$	(Abbas & Khan, 2007)
Salinity Index 6	$S6= ^{\mathrm{Red}\times \mathrm{NIR}}/_\mathrm{Green}$	(Abbas & Khan, 2007)
Soil-Adjusted Vegetation Index	$\mathrm{SAVI}=\frac{\mathrm{NIR}\;-\;\mathrm{Red}}{\left(\mathrm{NIR}\;+\;\mathrm{Red}\;+\;0.5\right)\times 1.5}$	(Huete, 1988)
Normalized Difference Vegetation Index	$\mathrm{NDVI}=\frac{\mathrm{NIR}\;-\;\mathrm{Red}}{\mathrm{NIR}\;+\;\mathrm{Red}}$	(Khan et al., 2005)
Canopy Response Salinity Index	$\mathrm{CRSI}=\sqrt{\frac{\mathrm{NIR}\;\times \;\mathrm{Red}\;-\;\mathrm{Green}\times \mathrm{Blue}}{\mathrm{NIR}\;\times\; \mathrm{Red}\;+\;\mathrm{Green}\;\times\; \mathrm{Blue}}}$	(Scudiero et al., 2015)
Brightness Index	BI = $\sqrt{{{\mathrm{Red}}^{2}+{\mathrm{NIR}}^{2}+\mathrm{Gr}}^{2}}$	(Khan et al., 2005)
Salinity ratio	$\mathrm{SR}=\frac{\mathrm{Green}\;-\;\mathrm{Red}}{\mathrm{Blue}\;+\;\mathrm{Red}}$	-
Vegetation Soil Salinity Index	$\mathrm{VSSI}=\left(2\times \mathrm{Green}\right)-5\times (\mathrm{NIR}+Red)$	(Dehni & Lounis, 2012)
Normalized Difference Salinity Index	$\mathrm{NDSI}=\mathrm{Red}-\mathrm{NIR}/\mathrm{Red}+\mathrm{NIR}$	(Khan et al., 2005)

Principal component analysis (PCA) is a statistical image enhancement technique commonly used for spectral transformation and reduction of redundancy information in datasets. The method allows the transformation of the original dataset into a smaller non-correlated which explains most of the total variation of the initial dataset. The set of PCs represents most of the information, and it is easier to use and analyzed for producing usable results. The PCA has been widely used in soil salinity assessment in a variety of ways (Hihi et al., 2019). For example, recently Abdelaal et al. (2021) performed PCA for the assessment and mapping of management zones in salt-affected soils of an arid region based on soil’s physical and chemical properties. Their results demonstrated the effectiveness of PCA for the identification of statistical differences in physical and chemical soil properties of salt-affected soils. In our study, the PCA was performed by using 13 variables (salinity and vegetation indices from the RS data) for the main components’ exportation (Abbas & Khan, 2007; Abdi & Williams, 2010). The PC1 and PC2 factors, which account for 99.9% of the total variance, were used to develop the probability model. For the PCA analysis implementation, the ArcMap GIS software was used.

### Soil salinity mapping

The model of soil salinity in the agricultural land (croplands) of Lesvos was developed using nine independent variables and a dependent variable using regression analysis technique (FAO, 2020). The spatial distribution of salt-affected soils came through the development of the probability model. For the probability model development and the assessment of the spatial distribution of salt-affected soils, the logistic regression analysis was applied (Kumar et al., 2019).

#### Logistic regression analysis

Logistic regression (LR) is a regression modeling technique defined as a process of modeling the probability of a discrete outcome by investigating the non-linear effect of a dependent categorical variable on the action of many independent variables. LR is applied to analyze the possible dependence of a response variable on more than one explanatory variable. In LR, the probability (percentage) of the occurrence of the two categories in relation to the independent variables which function as explanatory factors in relation to the dependent variable is examined (Hosmer and Lemeshow, 2001; Hosmer et al., 2013). LR model can support either categorical or continuous variables with or non-normal distribution. Binary logistic regression (BLR) is a binomial equation in which the response variable *Y* is the result of one of two outcomes such as event/present. The general expression of LR is given by the equation below:

1 $$P=\frac{1}{1+{e}^{-z}}$$

2 $$z={b}_{0}+\textstyle\sum_{i=1}^{n}{b}_{i}{x}_{i}$$

where *P* is the probability of the event occurrence, *z* is a linear combination of the independent variables, *b*_0_ is the model intercept, *b*_1_*…b*_*i*_ is the regression coefficient for explanatory variable *I*, *x*_1_*…x*_*i*_ is the explanatory variable *if*.

LR analysis was used herein to estimate the occurrence of soil salinity in the croplands of Lesvos according to independent factors (physical factors related to salinity formation in the soil). The LR method has been applied in many environmental studies (Lee, 2005; Nandi & Shakoor, 2010; Sarkar & Mishra, 2018). Moreover, it has also been used successfully in previous studies aiming at to identifying the distribution of salt-affected soils and assess soil salinity risk maps (Apel et al., 2020; Kumar et al., 2019). The development of the LR probability model follows a binary distribution, and the measurements of the dependent variable of the soil data were coded in values of 0 and 1. Two categories, saline and non-saline soils, with a threshold of 4 dS m^−1^ were used, based on the classification USSL Staff (1954) system. The encoding of the dependent variable for the regression analysis was performed according to the spatial decision support system (USSL Staff, 1954) for the determination and evaluation of saline soils based on EC_e_ using as threshold the value 4 dS m^−1^. For EC_e_ < 4 dS m^−1^, soils were considered non-saline and coded as 0 and for soils with EC_e_ > 4 dS m^−1^ that considered to be affected by salts and therefore were coded as 1.

According to Richards (1954)—and as summarized in Table 3**—**for the distribution of saline soils, the electrical conductivity of saturation paste is defined as EC_e_ > 4 dS m^−1^ with exchangeable percentage of sodium ESP < 15% and pH < 8.5. Sodic soil’s electrical conductivity is defined with low EC_e_ < 4 dS m^−1^ and high exchangeable sodium percentage ESP > 15% and pH values (pH > 8.5). It is noted that for EC_e_ measurements are taken and usually reported at a standard temperature of 25 °C and measured in mS/cm. However, the field measurements of the available soil data within the study region showed an ESP value of < 6 in 98% of all field measurements and a pH > 8.5 for only 2.6% of the soils. Therefore, EC_e_ was used as a dependent variable for the determination of saline soils and the development of the model.

**Table 3:** Salt-affected soils classification systems according to United States Department of Agriculture (USSL Staff, 1954)

	Soil properties		
Classification	EC_e_ dS m^−1^	ESP	pH
Saline soils	> 4	< 15	< 8.5
Sodic soils	< 4	> 15	> 8.5
Saline–sodic soils	> 4	> 15	< 8.5

All factors were normalized using the min–max method prior fitting regression analysis due to differences in measuring scales among the datasets. The LR modeling was performed in the SPSS IBM Statistics processing package using the block model, where a probability threshold higher than 0.5 was set for salt-affected soil and if the probability is less than 0.5 as absence so 0. In the applied method, all variables are considered from the model regardless of whether they are statistically significant in relation to the dependent variable. Default settings of cut-off value (0.5) and maximum number of iterations (20) was used. The model was derived through the logistic regression equation coefficients from the final stage output of the analysis (Table 5). Equation (3) was applied in ArcGIS software through the raster calculator tool in order to compute the probability of salinity in soils, chosen, as it includes the basic exponential function 10 (Exp10) to calculate the logistic regression equations as shown:

3 $$\begin{aligned}{P }_{({\mathrm{EC}}_{\mathrm{e}})}=& 1.0 / (1.0 +\mathrm{ Exp}10 (-\left(0.212 - 25.715 \times \mathrm{DEM} \right. \\ & + 0.072 \times \mathrm{ TWI} + 2.673 \times \mathrm{ slope }+ 17.220 \\ &\times \mathrm{CNBL} + \mathrm{plan curvature} \times 0.0001 \\&+ 5.666 \times \mathrm{min temperature}-1.957 \\ &\times \mathrm{max temperature} + 6.771 \times \mathrm{rainfall}\\ &\left. -10.658 \times \mathrm{ PC}1-1.942 \times \mathrm{PCA}2\right))\end{aligned}$$

The spatial join tool was subsequently applied so that each polygon representing a specific agricultural area receives a specific estimated probabilistic value for occurrence of soil salinity. By spatially joining the attributes based on their spatial relationship (i.e., latitude\latitude), a layer of joined features spatially related is generated.

#### Model validation

The logistic regression model prediction accuracy of saline soils was evaluated by calculating the receiver operating characteristic curve (ROC). The ROC is a fundamental tool for analyzing the performance of a model, plotted in a two-dimensional graph using true positive (sensitivity) rate on *y*-axis and false positive rate (1—specificity) on *x*-axis. The area under curve (AUC) value represents the quality of the probability model, describing its ability to predict the appearance or non-appearance of saline soils. In the ROC method, the value in the area below the curve between 0.5 and 1 is used to evaluate models’ accuracy (Nandi & Shakoor et al., 2010). If the AUC value is close to 1, high accuracy of the probability model is indicated (Fawssett, 2006). For the validation, 220 sample points were randomly created and extracted within the study area polygons. The ROC methods were applied to 220 independent EC_e_, used exclusively for the results validation of the analysis and therefore were not included for the development of the probability model.

### Soil organic carbon map

**Fig. 4:**
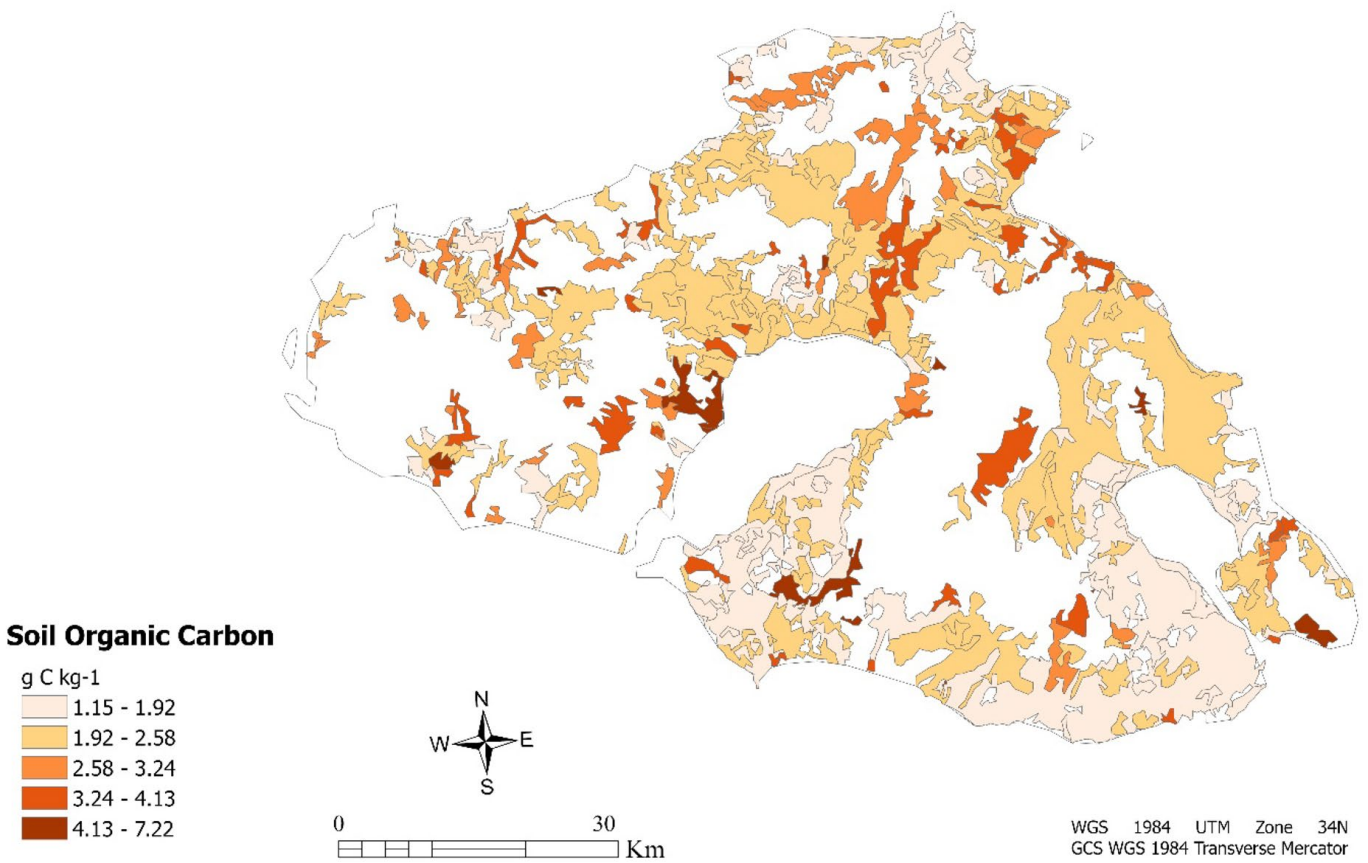
Soil organic carbon map of Lesvos in agricultural areas

A further study objective has been to investigate the spatial correlation between saline soils, as derived by the logistic regression model and the soil organic carbon stocks in the cropland areas of Lesvos. For this purpose, the pixel size was reduced using bilinear resampling method during the data pre-processing. The data was adjusted to the boundaries of the agricultural areas of Lesvos as formed by the Corine Database in the Corine Land Cover (CLC) 2018 program. Specifically, from the CLC map obtained, were selected 431 polygons representing agricultural areas. The spatial join tool was implemented for each polygon which represents a crop area receives a value of SOC. In the following figure (Fig. 4), the SOC content is presented only for the agricultural areas of the study area.

#### Spatial correlation between soil salinity and SOC

To understand the spatial trends in the data within the studied area, hotspot analysis (Getis-Ord Gi*) was also implemented among the Moran’s I global and local indicators. The correlation between the two soil parameters was performed after examining the autocorrelation values of the two variables. Hotspot analysis (based on Getis-Ord Gi* statistic) and the cluster and outlier analysis (based on Anselin local Moran’s I) were used to analyze the distribution patterns. The Global Moran’s I reveals a general pattern within the data distribution while the local Moran’s I identifies the location of these spatial patterns (i.e., low or high clustered values). Analysis of the spatial distribution patterns of each variable values allows assessing quantitatively the homogeneity and the differentiation in their spatial variation. In addition to estimating point densities in each area, hot spot analysis techniques also measure the extent to which point events interact to understand spatial patterns.

In our study, in calculating the Local Anselin Moran’s I, the *Inverse Distance* method was used. This method is suitable for continuous data or for modeling processes where the closer two adjacent features are in space, the more likely they are to interact or influence each other. As the distance method *Euclidean Distance* was selected, as it uses the straight line in connecting two points A and B. For the *Threshold Distance* parameter, the values of 1100 m and 2500 m were chosen for SOC and saline soils variable respectively, following results of the applied Global Moran’s I. Hot spot analysis method (Getis-Ord Gi*) was implemented through the optimized hot spot analysis tool which is an upgraded version of hot spot analysis as through automated procedures, the tool determines an appropriate scale of analysis using the distribution of weighted characteristics, makes corrections for spatial dependency testing with the False Discovery Rate (FDR) correction method, and determines the appropriate settings that will produce optimal results.

## Results

### Probability model results

In this study, factors related to salt-affected soils formation such as geomorphology, moisture, and other soil characteristics such as topographic indices, rainfall, temperature, and RS indices were used to estimate the spatial probability of salinity as well as to evaluate the distribution of soil salinity. For the topography of the study site, the elevation, slope, topographic wetness index, plan curvature, as well as channel network base level were used. Average minimum and maximum temperature and annual precipitation were used as climatic parameters. Various vegetation and salinity indices namely SI (1), SI (2), SI (3), SI (4), SI (5), SI (6), NDVI, NDSI, SAVI, SR, CRSI, BI, and VSSI were also added to the model. Through the model implementation, the spatial distribution of probability ranges of soil salinity in croplands of Lesvos obtained.

Results of the soil salinity predicted by the model indicate that the overall accuracy of the model to predict whether the soil is classified as saline is 97.1%. The statistical significance test of the model (Omnibus test) was found as ${\chi }^{2}$(9) = 52.63 and at *p* < 0.05, which implies a statistically significant finding. The coefficients of precipitation, annual temperatures, as well as soil geomorphology were significant to the development of the regression logistic model and to the prediction of the result as revealed from the [Exp (b)] statistic values of factors. Table 4 depicts the magnitude of the dependence of the dependent variable that can be interpreted by the variables acting as independent factors. The coefficient of DEM variable is negative indicating that elevation is negatively related to soil salinity in contrast to channel network base level which is positively related to salinity occurrence. The coefficient Nagelkerke *R*2 showed that the prediction model interprets 11.3% of the variance of the dependent variable from the independent variables. The *R*^2^ value is expected to be low, as Nagelkerke values usually take low values in logistic regression even in models where the parameters show a strong correlation with the result (Hu et al., 2006).

**Table 4:** Summary of logistic regression model and derived coefficients used for the evaluation of soil salinity model

**Model summary**	**-2 Log likelihood**	**Cox & Snell *R* square**	**Nagelkerke *R* square**
	455.628^a^	.026	.113
**Factors (independent variables)**			**Coefficient**
Elevation			−25.715
Topographic Wetness Index			.072
Slope			2.673
Plan curvature			.000
Channel network base level			17.220
Temperature min			5.666
Temperature max			−1.957
Precipitation			6.771
Principal component (PC1)			−10.658
Principal component (PC2)			−1.942
Constant			.212

^a^Estimation terminated at iteration number 8 because parameter estimates changed by less than .001

In evaluating the accuracy of a probabilistic model, the ROC curve method was used (Nandi & Shakoor, 2010). The ROC curve, which indicates the model fit quality, showed good results, suggesting that the model responds effectively to the data with area below the curve (AUC) of 0.73 (Table 5).

**Table 5:** ROC analysis results and area under the curve

**Area**	**S.E.**^**a**^	**Asymptotic Sig.**^**b**^
.729	.073	.001

^a^Under the nonparametric assumption

^b^Null hypothesis: true area = 0.5

LR was applied for the development of the saline soil probability model. The equation with the regression coefficients (Table 4) for the model development was executed after the logistic regression analysis applied. The values for the distribution of the probability of saline soils were calculated for croplands of Lesvos. The spatial distribution of the saline soils obtained where croplands have values close to 1, indicating a high probability of salinity with (EC_e_ > 4 dS m^−1^).

**Fig. 5:**
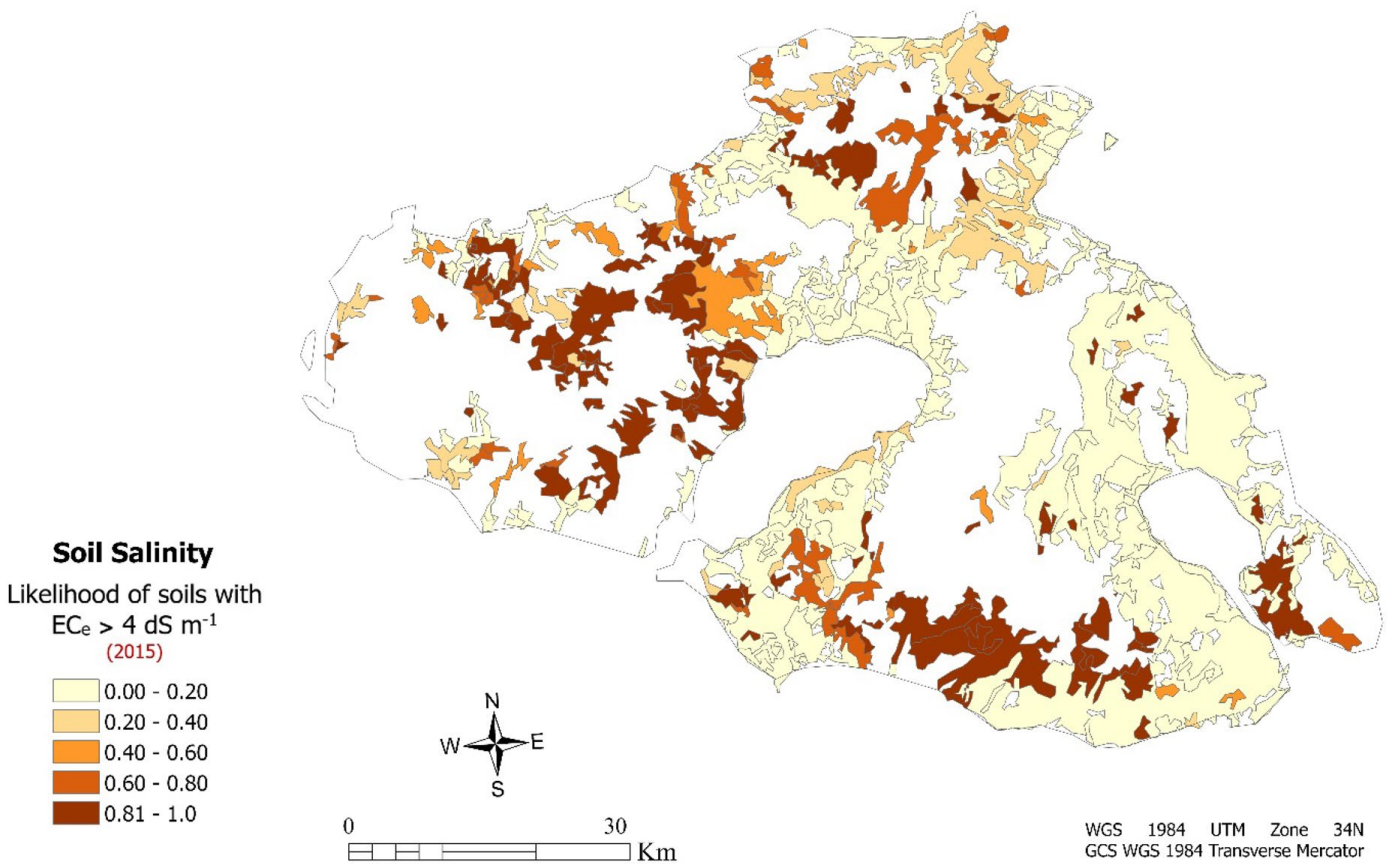
Model predictions indicating the likelihood of soil salinity at the depth 0–30 cm for the agricultural areas within Lesvos Island in 2015

However, for better interpretation and analysis of the results from the probabilistic model, the value expressing the probability is classified into five classes in the interval [0, 1]. In each polygon of 431 total polygons, which represents a specific land under agricultural exploitation (such as crop lands), a probability value is given of whether the crop occurs saline or not. Conclusions can be drawn regarding the interpretation of the distributed probabilities and the percentage covered by the agricultural areas with high probability of occurrence of saline soils, considering the intermediate values as presented in the following map (Fig. 5).

According to the logistic regression model results, most of agricultural land which covers about half of the total area (almost 55%) represent areas with absent or very low probability for salinity occurrence. Although a large-scale area of 169.51 km^2^ (23.7%) in extensive areas located in the western and SE part of the island are characterized by high probability (*p* > .80) for soil salinity occurrence (Table 6).

**Table 6:** Probability ranges of salt-affected soils (EC_e_ > 4 dS m^−1^) in Lesvos Island

**Probability**	**Area (**${\mathbf{km}}^{\varvec2}$**)**	**Area (%)**
0–.20	501.23	54.70
.20–.40	74.4	8.60
.40–.60	32.44	6.00
.60–.80	49.22	7.00
.80–1	169.51	23.70
Total	826.80	100

In the agricultural soils of Lesvos, soil salinization occurs as a result of both human and climatic factors. The main drivers of soil salinity are the relatively low percentage of annual rainfall, the high rates of evapotranspiration, and high index of drought. However, the processes of soil salinity are accelerated due to irrational and unsustainable soil management practices. Soils with a high probability of salinity are located in croplands of the western part, where there is a smaller amount of rain falling, with a decrease from east to west of over 45% (Kosmas et al., 1999). In our study area, crops that showed the highest probability and may be potentially affected by soil salinity are located in orchards with olive trees. Except for olive groves, non-irrigated areas are also prone to the occurrence of salinity and have shown a high probability of salinization, as a result of seasonal rainfall and dry-thermal climatic conditions (Shahid et al., 2018).

### Spatial correlation between SS and SOC

Indices of spatial autocorrelation, such as the Global Moran’s I and Anselin local Moran’s I and the Getis-Ord G statistic which were used in this study, allowed a comparison of the soil parameters (Table 7). Indicators of global and local spatial association were estimated to detect the different aspects of spatial correlation between the soil salinity and the SOC content. Global and local Moran’s I indices applied for the identification of spatial autocorrelation and local patterns of the values. The Anselin Moran’s I allowed the detection of both positive and negative spatial correlations of data values; while the Gi* statistic can distinguish the clustering of high and low values around the region (Scrucca, 2005).

**Table 7:** The positive spatial autocorrelation between the values of the two variables derived from Global Moran’s I index indicates spatial clusters of high and low values of soil salinity and organic carbon parameter

**Soil property**	**Index**	**Index value**	***z*** **score**	***p*** **value**
Soil salinity	Global Moran’s I	0.490	110.428	0.0000
Soil organic carbon	Global Moran’s I	0.360	53.139	0.0000

**Fig. 6:**
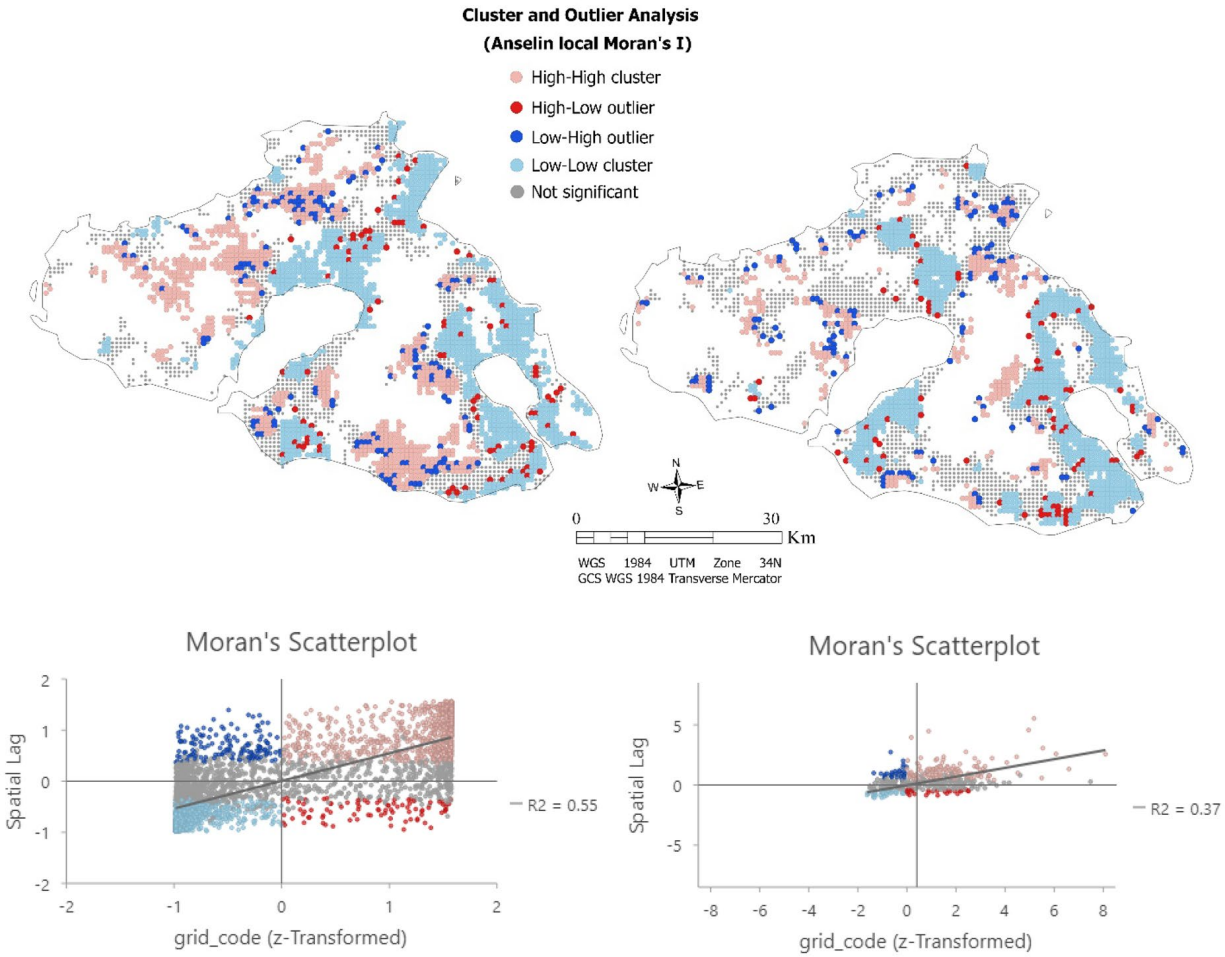
The cluster and outlier analysis of Anselin Local Moran’s I index derived the cluster map and scatterplot for (left) saline soils variable and (right) SOC variable

The cluster map and the local Moran’s scatter plot provide a classification of spatial association into five classes, corresponding to the location of the points in the four quadrants of the plot (Fig. 6). For spatial clusters representing low values nearby low values (LL) or high value nearby similarly high values (HH) is marked with lighter blue and red, respectively. Spatial patterns which represent regions with low values nearby high values (LH or HL) and vice-versa are called outliers and are marked with dark blue and red, respectively. The spatial distribution of the values among the two soil parameters revealed interesting spatial patterns, particularly so in case of probability ranges of soil salinity. Indeed, as can be observed, a positive spatial autocorrelation is noted that in both cases of soil parameters with low values surrounded by nearby low values (clustered) accounting for 33.9% for salinity and 30.8% for SOC (Fig. 6).

In northern regions, clusters with high values of salinity probability occurred where clusters of low SOC values appeared (Table 8). Respectively, in the eastern part, clusters of high values in salinity probability are observed in regions with clusters of low values in SOC. In regions where both values of similar low or high clusters in salinity probability and SOC are appeared, the outliers indicated the existence of dissimilar nearby values. Most of the outliers are observed inside in region with clustered patterns. Indeed, HL outliers in the eastern and southern part of the island and LH outliers mainly in central and western part can be linked to regions where clusters with statistically significant values of two variables occurred. High soil salinity probability values are clustered in agricultural areas of western part in land principally occupied by agriculture with small areas of natural vegetation and in non-irrigated arable land. In central and southern rural areas, high clusters correspond to arable land with significant areas of natural vegetation and extended areas of olive groves. Respectively, the significant low values of salinity probability clustered in central agricultural areas correspond in areas with sclerophyllous vegetation and complex cultivation patterns. An extended cluster of low values for both soil parameters shows a correspondence to a large land of olive groves, which also caused the effect of the HL outliers’ appearance. On the other hand, the southern and central clusters of low values in SOC are located in extended areas with non-irrigated arable land and olive groves.

**Table 8:** Percentage of clustering patterns for soil salinity and soil organic carbon point metrics by Anselin local Moran’s I

**Soil property**	**Index**	**HH**	**LL**	**HL**	**LH**	**NS**	**Total**
		(%)	(%)	(%)	(%)	(%)	
**SS**	Anselin local Moran’s I	25.9	33.9	2.8	4.4	33.0	100
**SOC**	Anselin local Moran’s I	12.3	30.8	2.5	4.2	50.1	100

According to the local Moran’s I scatterplots (Fig. 6), an overall clustered pattern of positive spatial autocorrelation is indicated, which is statistically significant for both soil parameters of soil salinity probability (Moran’s I = 0.55) and soil organic carbon (Moran’s I = 0.37).

**Fig. 7:**
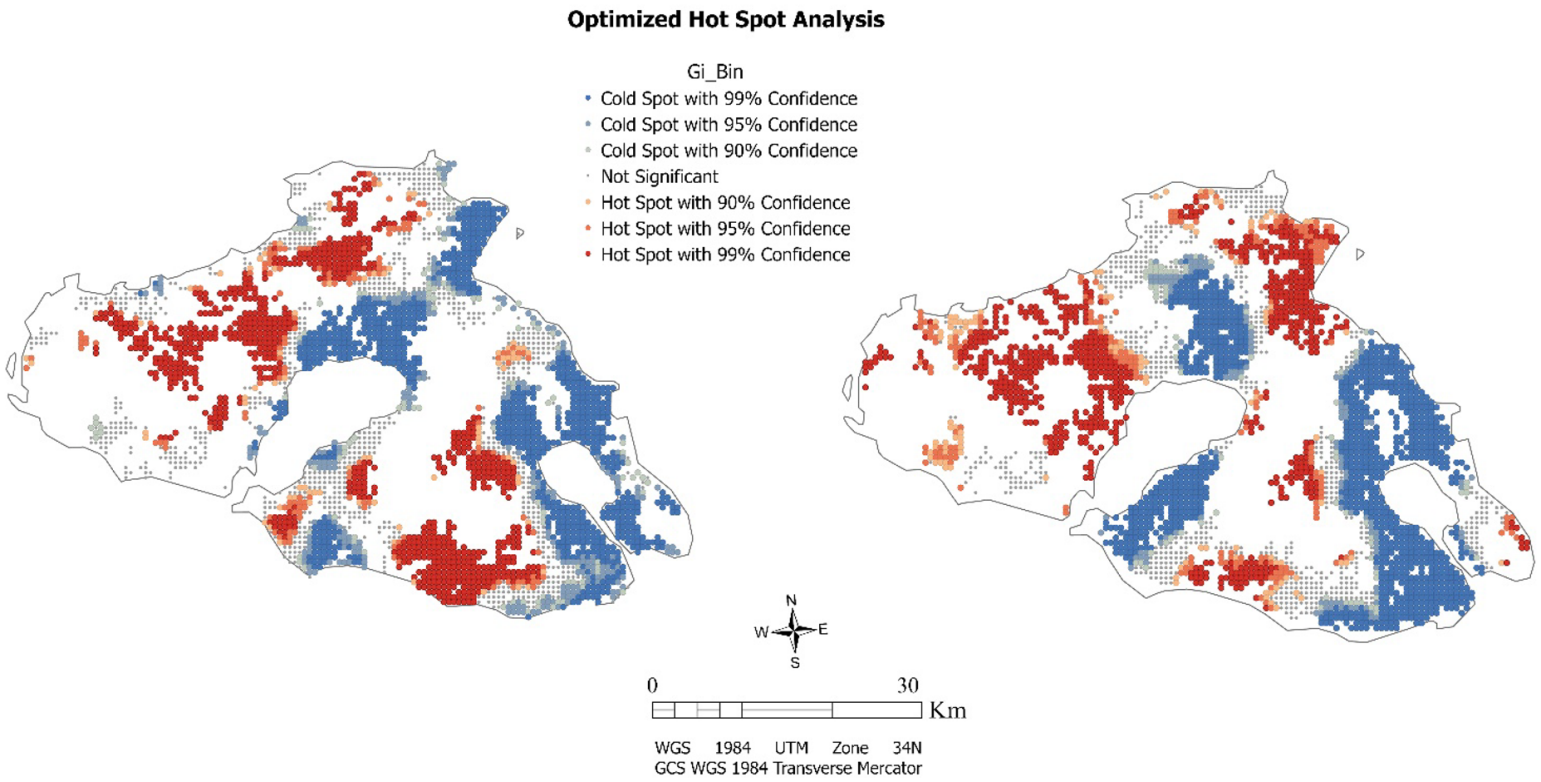
Hot spot analysis (Getis-Ord Gi*) for variables of soil salinity probability (left) and SOC (right)

The Getis-Ord Gi* statistic was calculated using the hot spot analysis (Getis-Ord Gi*). Statistically significant spatial patterns are identified from the classification of local Getis Gi* indicators within the study region. Hot spots of salinity are observed in the southeastern and the central parts of the study area on the contrary of SOC that cold spots are observed mainly in the southeastern part. Correspondingly, this phenomenon appears to be the case in the northern section. For the probability of soil salinity occurrence values, the clusters where there is a concentration of high values appear in central and northern part of the island, where they are mainly covered by olive groves as well as in areas in the eastern part in lands principally occupied by arable land. Clusters of low values are observed along the eastern and southeastern parts in the region where complex cultivation systems and agricultural land with significant areas of natural vegetation dominate. Conversely, the concentration of low values of soil organic carbon reserves extends mainly along the central and southern part of the island. High SOC levels are found in the northern part where mostly natural and sclerophyllous vegetation can be found. This also appears to be the case in the eastern and southeastern parts of the island. However, in some cases, SOC levels can be found to be inversely high in areas with a high probability of being saline. This could be linked to the heterogeneity of the croplands which consists of significant areas of natural vegetation. It is noted that the local Moran’s I index revealed outliers of LH and HL values in many areas with similarities in clustered values of both soil parameters. In the following figure are presented the hotspots analysis results concerning the examined variables (Fig. 7).

## Discussion

In this study, a probabilistic method based on logistic regression (LR) has been developed for mapping the spatial distribution of soil salinity in cropland areas of Lesvos, adopting geoinformation technologies. The LR method has been applied previously in a wide range of environmental studies including, for example, soil erosion and landslides (Sarkar & Mishra, 2018). LR has also been successfully used before to identify and classify salt-affected soils. For example, Kumar et al., (2019) using Landsat 8 OLI multispectral satellite data developed a model to identify salt-affected soils, and in comparisons, they performed versus corresponding field survey data reported the model to somehow overestimate the occurrence of saline soils in cropland areas.

The model developed in our study showed quite satisfactory results in predicting saline soils in agricultural areas. The Nagelkerke *R*^2^ coefficient indicates that the probability model interprets 11.3% of the variance of the dependent variable from the independent variables. The climatic factors of annual temperatures and rainfall added in the model (annual temperature—*p* < .05 and rainfall—*p* < .005) as well as the topography (DEM—*p* < 0.001 and channel network base level *p* < .008) were statistically significant and contributed as factors in the development of the model and the evaluation of the results. In contrast to some predictors such as the topographic curvature index (Sig. .954) and the second principal component (Sig. .254) were not found to be statistically significant in the model, although the PC1 was a statistically significant factor in the analysis (*p* < .009). The ROC curve was used to verify the model’s prediction accuracy. The model showed good adaptability and responds well to the data with the area value under the ROC curve was found to be (AUC) .73.

Similar studies that have been conducted previously at different settings have also underlined the influence of the aforementioned predictors in soil salinity. For example, Nie et al. (2021) evaluated the effectiveness of terrain factors such as DEM, slope, and TWI using kriging regression techniques in order to predict the extent of secondary salinization, in a setting of northeast China. Later, Sahbeni (2021) used field data and spectral salinity and vegetation indices derived from Landsat 8 OLI for developing soil salinity prediction maps in the Great Hungarian Plain using regression modeling. This study demonstrated the efficiency of the elevation factor which was statistically significant for soil salinity prediction, with a *p* value equal to 0.002, as in the case of this study.

The salinization of soils is a significant threat to the environment and to soils’ fertility and quality. In addition, soil salinization reduces crop yields and food security globally (Shrivastava & Kumar, 2014; Gorji et al., 2015). As the climatic conditions of the study area promoted the formation of soil salinization, agricultural management practices and irrigation supply systems should be adjusted in order to mitigate the effects of the reduction of agricultural production and the fertility of soil resources. The saline soils in the agricultural areas of Lesvos are mainly due to the relatively low percentage of rainfall, the high index of bioclimatic drought, and the unsustainable management practices applied in agricultural areas (Kosmas et al., 2000, 2002). Specifically, in the western part of the island, a smaller amount of rainfall is recorded. It is observed that croplands with a higher probability of soil salinity mainly occur in agricultural areas where the vegetation consists mainly of olive groves, which correspond to most of the island (Kosmas et al., 1999). There is also a high probability of saline soils occurring in non-irrigated arable land (mainly in the western and SE part), as a result of dry-thermal climatic conditions because of the increased evapotranspiration of soil (Shahid et al., 2018). Lastly, according to the results in the study area, it must be noted that agricultural areas corresponding to arable and non-irrigated arable land as well as olive groves are more prone to the soil salinization process.

Several studies have also attempted to study SOC content by applying spatial analysis and GIS methods (Bhunia et al., 2015; Bhardwaj et al., 2019; Zhang et al., 2020). In recent years, more studies have focused on the exploration of SOC stocks and saline soils, mainly in areas under agricultural development (Emran et al., 2020; Sakai et al., 2020). In our study, the spatial investigation of soil salinity and organic carbon stocks in croplands of Lesvos attempted, using geoinformation technologies. Cluster mapping techniques which were used to establish the spatial distribution of soil parameters, revealed that soils with high probability of salinity and high SOC content in these soils follow a different spatial pattern. High values of soil salinity probability are clustered mainly in areas where low values of SOC are clustered, or outliers occurred. Due to assumptions that high probability of soil salinity could potentially affect SOC content, outliers are expected to be found in regions where similarities in high and low clusters of two parameters occurred. As indicated in the obtained results, the two variables appear to be potentially correlated, which indicates that the soils with high probability of salinity occurrence may affect soil organic carbon content in those soils. Application of the geospatial analysis techniques of the two soil parameters as performed in this study suggested the negative effects of soil salinity in areas where an increased probability occurs in soil organic carbon content. Notably, previous studies have indicated that salt-affected soils tend to minimize carbon dioxide inflows and contain a limited amount of SOC, while increasing its carbon release rates into the atmosphere through soils degradation (Setia et al., 2011, 2013; Wong et al., 2010; Zhao et al., 2017).

## Conclusions

In this study, the likelihood of salinity for agricultural areas in Lesvos was estimated through the development of a probabilistic model using geoinformation technologies including RS and GIS. In addition, an attempt was made to investigate the relationship between SOC and saline soils using geospatial data analysis methods. The main conclusions drawn are summarized below:

(i) The logistic regression model predicted correctly 97.1% of the observations. Nagelkerke *R*^2^ coefficient interprets 11.3% of the variance of the dependent variable from the factors set as independent. The Omnibus test fit test showed that the overall model is statistically significant with ${\chi }^{2}$(9) = 52.63, *p* < .005. The area under the ROC curve was (AUC) 0.73. Thus, this method can be used as an approximate method with efficient results for the prediction of salinity in agricultural soils and may be sufficiently applied in other areas with similarities in environmental conditions.
(ii) The western part of Lesvos island covers agricultural areas with the highest probabilities of soil salinity which may be associated with environmentally sensitive areas where salt-affected soils or soils prone to salinization have been recorded from previous studies (Kosmas et al., 1999, 2002). A total of ∼20% of the agricultural areas are characterized by a high probability of soil salinity (*p* > .80). Approximately, a total area of 169.51 km^2^ mainly in the western and south-eastern parts indicating high probability of soil salinity occurrence (with EC_e_ > 4 dS m^−1^) which indicates the need for further investigation of the results of this study.
(iii) Geospatial analysis findings suggested that a small variation of SOC content is shown in soils where an increased probability of soil salinity occurs. Different spatial distribution patterns of two variables indicated the negative spatial correlation patterns of the two soil parameters, as a result of the adverse effects of saline soils or soils prone to salinity in SOC accumulation.

Our study provides a methodological framework approach that has a promising potential to support decision making on agricultural land protection and agricultural planning in general. There are also numerous pathways that can be followed in taking this study further. Among the key priority ones include the development of models for the study of different variations of salts concentration in salt-affected soils and the quantification of the correlation between the examined soil parameters. Such models, if available, could significantly improve the interpretive capacity of the results for the study area as the two soil parameters are not constant and change in space and time.

## Data Availability

The data that supported this study is available from ELGO DEMETER, Greece. Restrictions apply to the availability of these data, which were used under license for this particular study only. Data has become available to the authors with the permission of ELGO DEMETER.
